# How I do it: lateral canthal web revision—single Z-plasty technique

**DOI:** 10.1186/s40463-022-00585-7

**Published:** 2022-09-16

**Authors:** James Fowler, Corey C. Moore

**Affiliations:** Department of Otolaryngology—Head and Neck Surgery, Schulich School of Medicine and Dentistry, St Joseph’s Healthcare Centre, Western University, 268 Grosvenor St, London, ON N6A 4V2 Canada

**Keywords:** Lateral canthal web, Z-plasty, Blepharoplasty, Revision surgery

## Abstract

**Background:**

Lateral canthal webbing is a known complication of blepharoplasty, which occurs when the lateral aspect of the upper blepharoplasty incision is taken below the equator of the lateral canthus. Removing excessive eyelid skin laterally can also result in a lateral canthal web. Currently, there is no standard approach for addressing this complication.

**Methods:**

Retrospective review of single surgeon practice between 2011 and 2019. All patients underwent revision surgery using the proposed single Z-plasty technique.

**Results:**

Twenty-three patients referred for lateral canthal web were included in the study. All patients had previous upper lid blepharoplasty, with the initial procedure occurring 8–63 months prior to the referral for revision. The majority of the blepharoplasties occurred in Ontario (n = 19), but some patients also underwent surgery in Alberta (n = 1), British Columbia (n = 1), and United States (n = 1). The initial surgeries were performed by a variety of specialities including plastic surgery (n = 16), otolaryngology (n = 4), ophthalmology (n = 2), and family medicine (n = 1). Following revision surgery using the single Z-plasty technique, all patients reported a subjective increase in functional and aesthetic satisfaction. No further revision surgery was required for any of these patients.

**Conclusion:**

The single Z-plasty technique is simple, robust, and could be easily incorporated into any cosmetic practice to address this complication of blepharoplasty.

**Graphical Abstract:**

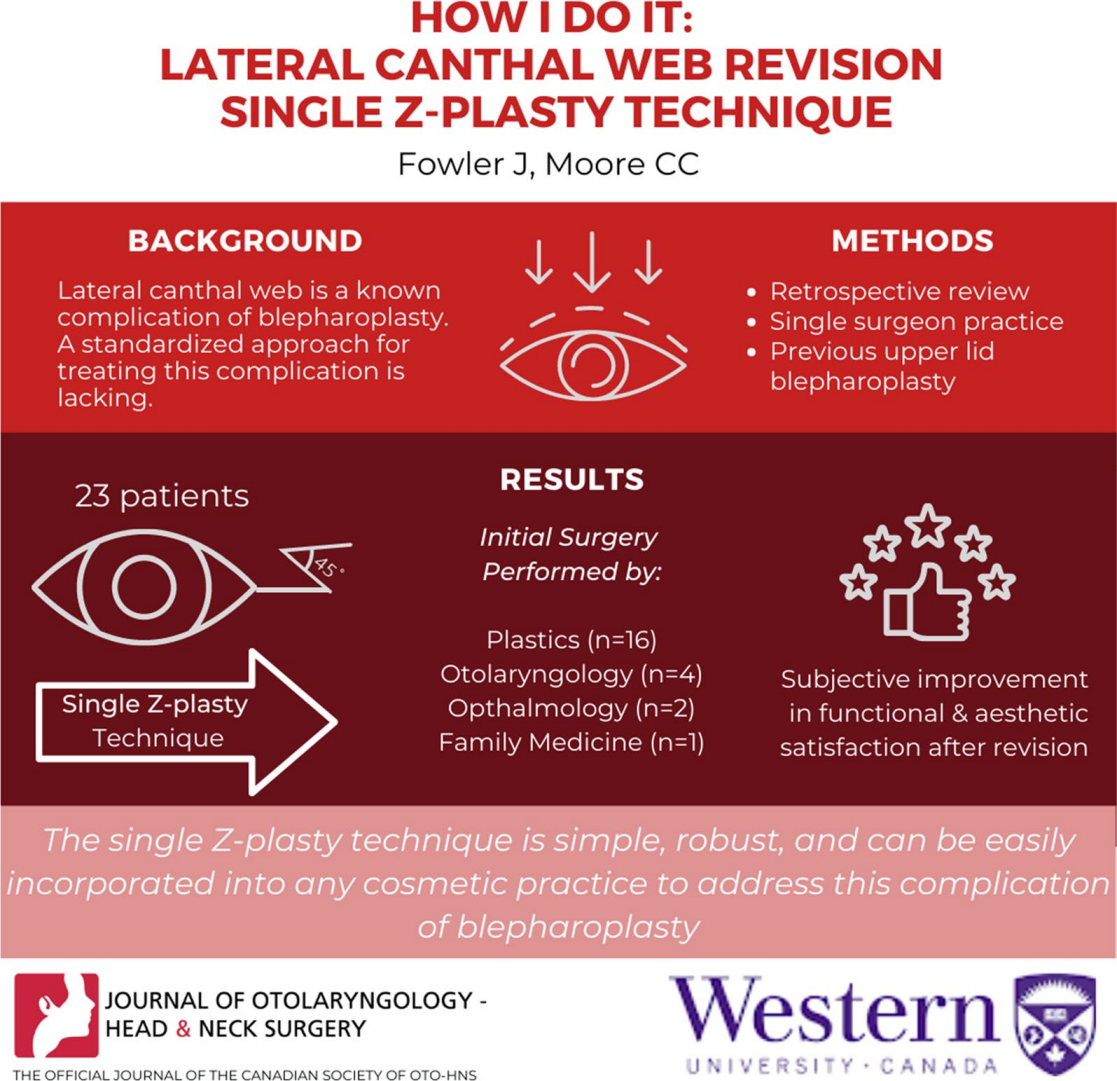

## Background

Upper eyelid blepharoplasty is a surgical procedure in which excess skin, fat, and/or muscle are removed from the upper eyelid to correct lid redundancy. The term was first coined in 1818 by Karl Ferdinand Von Graefe, who used the technique to reconstruct the eyelid post-cancer resection [1]. Since then, blepharoplasty has become one of the most commonly performed procedures in cosmetic surgery in the United States [2]. Blepharoplasty can be performed for both aesthetic and functional reasons. Vision impairment secondary to dermatochalasis, epiblepharon with lash ptosis, severe blepharochalasis, inflammatory disorders (Grave’s Disease), or eyelid trauma are the most common functional indications for blepharoplasty [3, 4]. Cosmetic indications include eyelid fullness, and eyelid asymmetry [5].

**Fig. 1:**
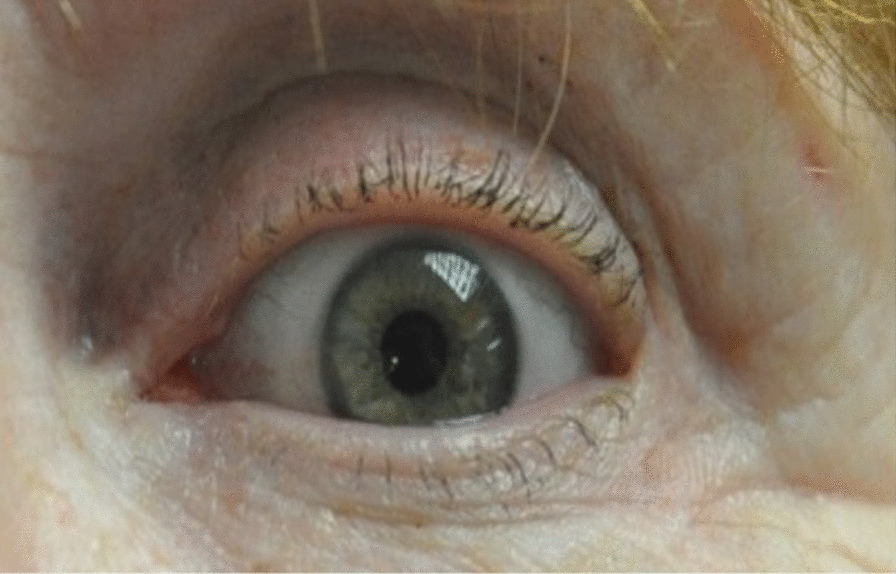
Left lateral canthal web

Overall, most patients are satisfied with the appearance of their eyelids post-blepharoplasty, but, as with all surgical procedures, it is not without risk of complication. Lid asymmetry, lagophthalmos, lacrimal duct injury, hematoma formation, medial canthal webbing, and wound dehiscence are all potential complications [5]. A less reported complication is lateral canthal webbing. A lateral canthal web occurs when the lateral aspect of the upper blepharoplasty incision is taken below the equator of the lateral canthus. It can also occur if excessive eyelid skin is removed laterally. During healing, scar contracture pulls the lax lower lateral eyelid skin superior and upper eyelid skin inferior, thus causing skin bridging across the lateral canthus (Fig. 1).

Simple excision of the lateral canthal web will not improve the skin bridge, and often makes it much worse. The senior surgeon (CM) has received many patient referrals for blepharoplasty revision, secondary to lateral canthal webbing. The purpose of this paper is to share our experience using our single Z-plasty technique for correcting this avoidable complication.

## Methods

A retrospective review was completed for patients referred to the senior author between 2011 and 2019. Patients who had a lateral canthal web, and underwent revision surgery, were included in this study.

### Surgical technique

**Fig. 2:**
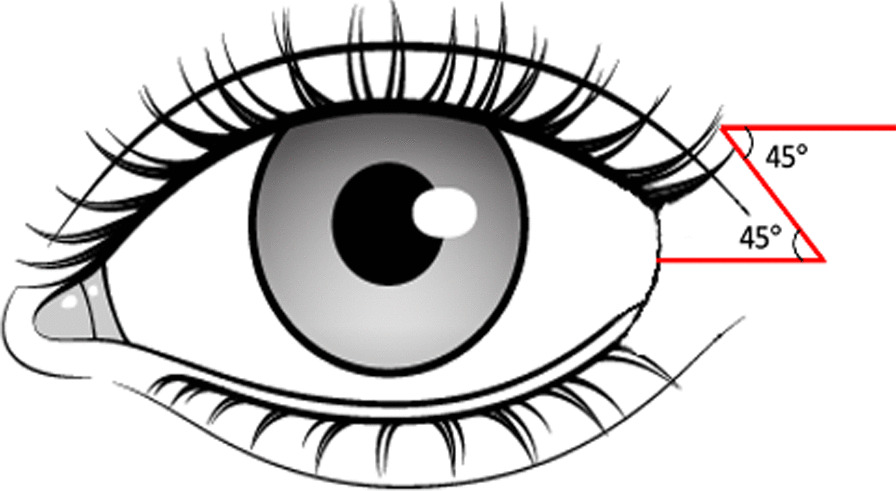
Positioning of z-plasty. The inferior limb is positioned across the skin bridge of the lateral canthus. Z-plasty limb lengths of approximately 1 cm with an angle of 45 between each limb

Our technique encompasses the use of a single z-plasty positioned with the inferior limb across the lateral cantal web. Each limb is approximately 1 cm in length, with an angle of 45 degrees between each (Fig. 2).

**Fig. 3:**
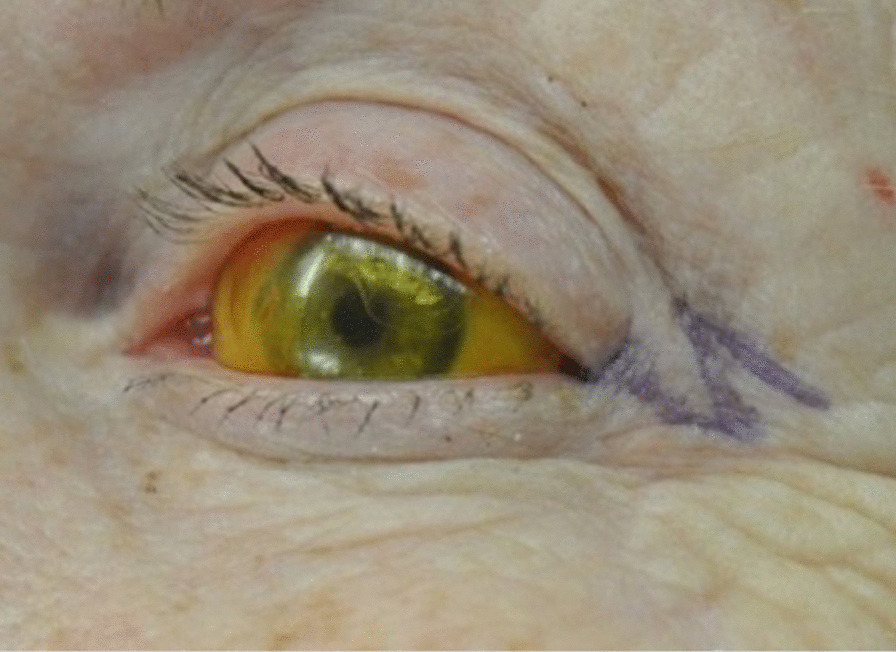
Pre-operative marking of the Z-plasty

**Fig. 4:**
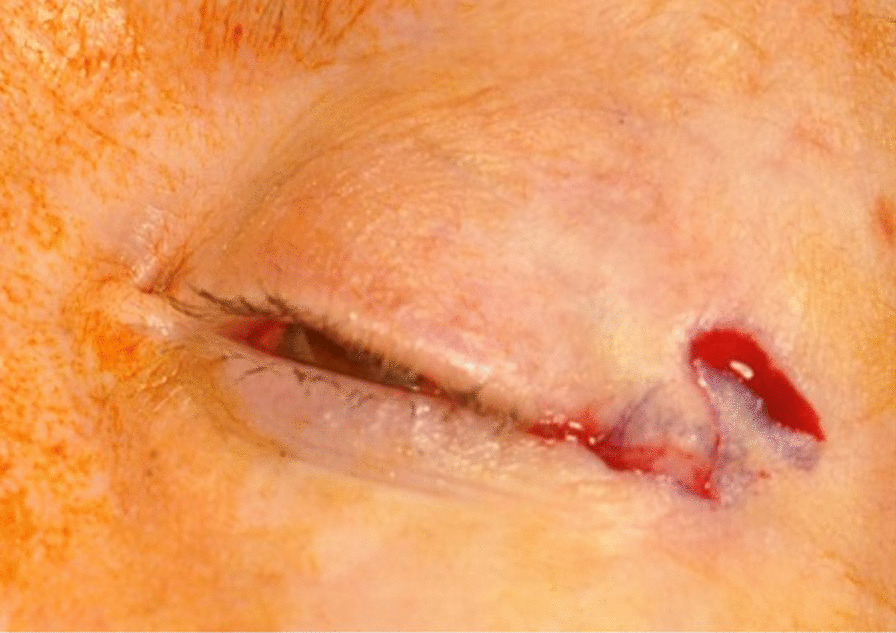
Z-plasty incision, starting at the lateral canthal web

**Fig. 5:**
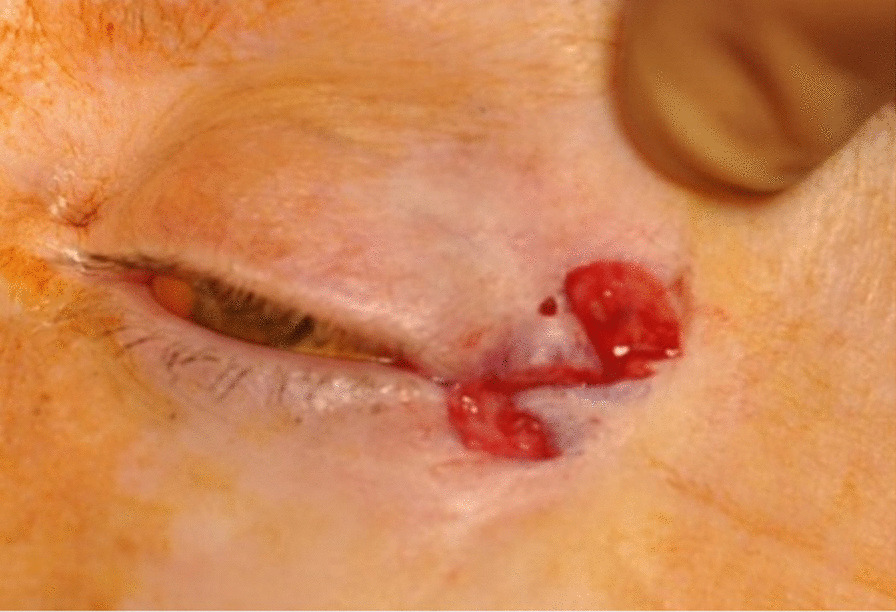
Transposition of the superior and inferior flaps of the Z-plasty

**Fig. 6:**
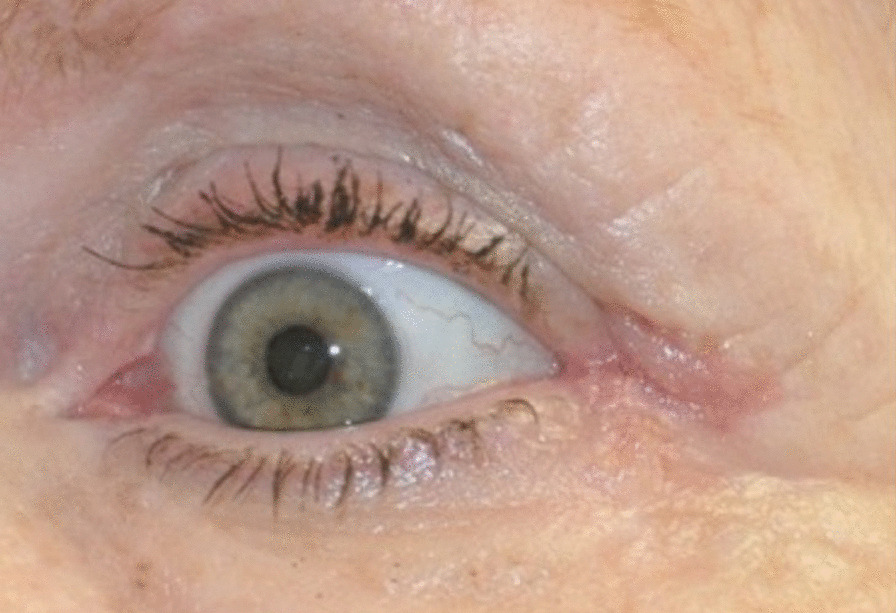
Lateral canthus post-operative day 10

The procedure begins by marking out the skin with the z-plasty positioned along skin bridge of the lateral canthus (Fig. 3). Approximately 2 ml of 1% xylocaine with 1:100,000 epinephrine is injected into the lateral canthus. The surgical area is then prepped and draped in a sterile fashion. The initial incision should be made along the lateral canthus (Fig. 4). The inferior triangular flap of the Z-plasty incorporates the skin of the upper eyelid that has scarred into the skin bridge of the lower eyelid. It is important to keep this portion of skin in the upper eyelid. The upper flap of the Z-plasty involves the superolateral orbital skin that will be transposed in between the upper and lower eyelid skin (Fig. 4). This flap acts as a spacer, allowing separation between upper and lower eyelid skin, thus decreasing risk of further canthal web formation (Fig. 5). Closure is performed using a single layer of 6.0 silk suture in an interrupted fashion. Mupirocin ointment is applied to the incision for 7 days. Sutures are removed 10 days post-operatively (Fig. 6).

## Results

Twenty-three patients were included in the study (19 females, 4 males). All patients had previous upper lid blepharoplasty, with the initial procedure occurring 8–63 months prior to the referral for revision. The majority of the blepharoplasties occurred in Ontario (n = 19), but some patients also underwent surgery in Alberta (n = 1), British Columbia (n = 1), and United States (n = 1). These initial surgeries were performed by a variety of specialities including plastic surgery (n = 16), otolaryngology (n = 4), ophthalmology (n = 2), and family medicine (n = 1). Seven patients had previous canthal scar revisions by the original surgeon with little success. For these patients, the primary attempt at revision occurred 13–21 months after the original surgery.

Upon referral, patient complaints were both functional and aesthetic. Functionally, 20 patients reported feeling of tightness or fullness along the lateral aspect of the eyelid. Six patients also reported visual hinderance, which manifested as restricted lateral visual fields. All 23 patients had lid asymmetry. Following revision using the single Z-plasty technique, all patients reported a subjective increase in functional and aesthetic satisfaction. No further revision surgery was required for any of these patients.

## Discussion

Blepharoplasty has become one of the most commonly performed procedures of cosmetic surgeons in North America. Lateral canthal webbing is a known, and avoidable complication of blepharoplasty. Precise pre-operative marking of the eyelid is crucial for success. A common pitfall is the inclusion of eyelid skin below the equator of the lateral canthus. This error will almost guarantee the formation of a skin web.

The technique of blepharoplasty is well documented throughout the literature, but unfortunately there is a paucity of research for revision surgery involving the lateral canthus. A review of the literature reveals only two studies that address lateral canthal webbing. The first was a case report by Lee et al. [6] This patient, a 71-year-old female, developed a lateral canthal web nine months after upper and lower lid blepharoplasty. In their study they used the del Campo Transposition Technique, which was initially used for repair of epicanthal folds. This technique transposes the skin laterally, thus increasing skin tension of the lateral canthus. Following the procedure, the patient reported functional and aesthetic improvements.

The second study was a retrospective review by Massry [7]. Their study involved a single surgeon who performed seven lateral canthal revisions using the Five Flap Technique between 2006 and 2010. This technique incorporates two skin transposition maneuvers, the Y-to-V advancement flap and two Z-plasties centred on each of the arms of the Y. All patients received three weekly injections of Kenalog starting three weeks after the surgery. Postoperatively, all lateral canthal webs were significantly reduced, and patients reported improved visual function.

Although both aforementioned techniques appear to resolve lateral canthal webbing, they lack simplicity, and robustness. Z-plasty is one of the most common skin transposition techniques, making our technique simple and easy to incorporate into any cosmetic surgical practice. The previous two studies are also of small sample size. To date our study is the largest on this topic, including 23 patients over an 8 year period, thus showcasing the longevity of our technique.

## Conclusion

A lateral canthal web is a known complication of blepharoplasty. It is both frustrating for patient and surgeon as there lacks standards for its correction. This paper presents our experience using the single Z-plasty technique to successfully correct lateral canthal webs. Although this technique is described to treat a complication of upper blepharoplasty, it can also be applied any webs (medial or lateral) secondary to periocular surgery. We believe this technique is a valuable tool, and should be learned by any surgeon who operates in the periocular region.

## Data Availability

All data generated or analysed during this study are included in this published article.

## Abbreviation

CM: Corey Moore
